# Subclones in B-lymphoma cell lines: isogenic models for the study of gene regulation

**DOI:** 10.18632/oncotarget.11524

**Published:** 2016-08-23

**Authors:** Hilmar Quentmeier, Claudia Pommerenke, Ole Ammerpohl, Robert Geffers, Vivien Hauer, Roderick AF MacLeod, Stefan Nagel, Julia Romani, Emanuela Rosati, Anders Rosén, Cord C Uphoff, Margarete Zaborski, Hans G Drexler

**Affiliations:** ^1^ Leibniz-Institute DSMZ-German Collection of Microorganisms and Cell Cultures, Braunschweig, Germany; ^2^ Institute of Human Genetics, Christian-Albrechts-University Kiel and University Hospital Schleswig-Holstein, Kiel, Germany; ^3^ Genome Analytics Research Group, Helmholtz Centre for Infection Research, Braunschweig, Germany; ^4^ Department of Experimental Medicine, Bioscience and Medical Embryology Section, University of Perugia, Perugia, Italy; ^5^ Department of Clinical and Experimental Medicine, Division of Cell Biology, Linköping University, Linköping, Sweden

**Keywords:** CD5, cell lines, CLL, clonal evolution, subclones

## Abstract

Genetic heterogeneity though common in tumors has been rarely documented in cell lines. To examine how often B-lymphoma cell lines are comprised of subclones, we performed immunoglobulin (*IG*) heavy chain hypermutation analysis. Revealing that subclones are not rare in B-cell lymphoma cell lines, 6/49 IG hypermutated cell lines (12%) consisted of subclones with individual *IG* mutations. Subclones were also identified in 2/284 leukemia/lymphoma cell lines exhibiting bimodal CD marker expression. We successfully isolated 10 subclones from four cell lines (HG3, SU-DHL-5, TMD-8, U-2932). Whole exome sequencing was performed to molecularly characterize these subclones. We describe in detail the clonal structure of cell line HG3, derived from chronic lymphocytic leukemia. HG3 consists of three subclones each bearing clone-specific aberrations, gene expression and DNA methylation patterns. While donor patient leukemic cells were CD5^+^, two of three HG3 subclones had independently lost this marker. *CD5* on HG3 cells was regulated by epigenetic/transcriptional mechanisms rather than by alternative splicing as reported hitherto. In conclusion, we show that the presence of subclones in cell lines carrying individual mutations and characterized by sets of differentially expressed genes is not uncommon. We show also that these subclones can be useful isogenic models for regulatory and functional studies.

## INTRODUCTION

Tumors evolve under selective pressures, including the inhibitory influences of anti-cancer drugs. Clonal evolution is an important topic in cancer research because it underlies development of heterogeneous, molecularly related tumors in one patient and may explain why relapsed samples are often genetically distinct from primary diagnostic clones [1–3].

Permitting functional analysis of oncogenes against a syngenic background, homologous cell lines would appear ideal model systems. However, little is known about the clonal composition of cell lines, notably whether they represent true pathologic subclones of the primary tumor rather than mere culture artifacts. The diffuse large B cell lymphoma (DLBCL) cell line U-2932 is an example of a cell line comprising subclones, with differential expression of > 100 genes, including the germinal center oncogenes *BCL6* and *MYC* [4]. The bimodal expression of various B-cell markers on U-2932 allowed flow-sorting of the subclones which – underlining their usefulness - let to the discovery that *BCL6* can drive expression of germinal center markers in DLBCL [5].

Here, we set out to examine how often cell lines consist of subclones. Immunoglobulin (*IG*) hypermutation analysis revealed that 6/49 (12%) B-lymphoma cell lines comprised subclones. We show furthermore, that bimodal cell surface marker expression can also be indicative of subclones. Thus, the chronic lymphocytic leukemia (CLL) cell line HG3 includes CD5^+^ and CD5^−^ subclones. We describe the clonal structure of this cell line in detail. The usefulness of isogenic subclones for the study of gene regulation was established by showing that *CD5* expression may be regulated at the level of transcription rather than by the alternative splicing mechanism reported hitherto [6, 7].

## RESULTS

### Immunoglobulin hypermutation analysis identifies cell line subclones

The gain of *IG* hypermutations marks an important stage in B-cell development, occurring in the dark zone of the germinal center. This process can proceed during lymphoma evolution leading to the rise of subclones with common and subclone-specific *IG* mutations. Therefore, we performed *IG* heavy chain (IGHV) hypermutation analysis to detect subclones using B-lymphoma cell lines as material.

*IG* rearrangements were determined in 59 cell lines by PCR analysis with primers specifically recognizing the different VH-JH rearrangements [8]. The PCR products were cloned and sequenced. With mutation levels higher than 2%, 49/59 B-lymphoma cell lines (83%) exhibited *IG* heavy chain hypermutations (Supplementary Table S1). Among hypermutated cell lines 6/49 (12%) consisted of subclones. In these 6 cell lines RAJI, OCI-LY7, SU-DHL-5, TMD-8, U-2932 and U-2940, > 3/10 sequenced bacterial clones (i.e. *IG* PCR products) exhibited subclone-specific mutations, confirming the presence of two or more clones in these cell lines (Supplementary Table S1).

Of cell lines with *IG* hypermutations 25/49 (51%) were DLBCL-derived. The remaining 24 (49%) represented Burkitt lymphoma (*n* = 9), mantle cell lymphoma (*n* = 1), multiple myeloma (*n* = 8), primary effusion lymphoma (*n* = 3) and Hodgkin disease (*n* = 3). Five cell lines showing interclonal IGHV variation (OCI-LY7, SU-DHL-5, TMD-8, U-2932, U-2940) were DLBCL-derived. The only non-DLBCL cell line with subclones was the Burkitt lymphoma cell line RAJI (Supplementary Table S1).

### Bimodal surface marker expression as indicator for subclones

*IG* hypermutation analysis was performed as the method of choice to screen B-lymphoma cell lines for subclones. To assess whether other cell lines might also comprise subclones, we performed immunophenotyping analysis.

**Figure 1:**
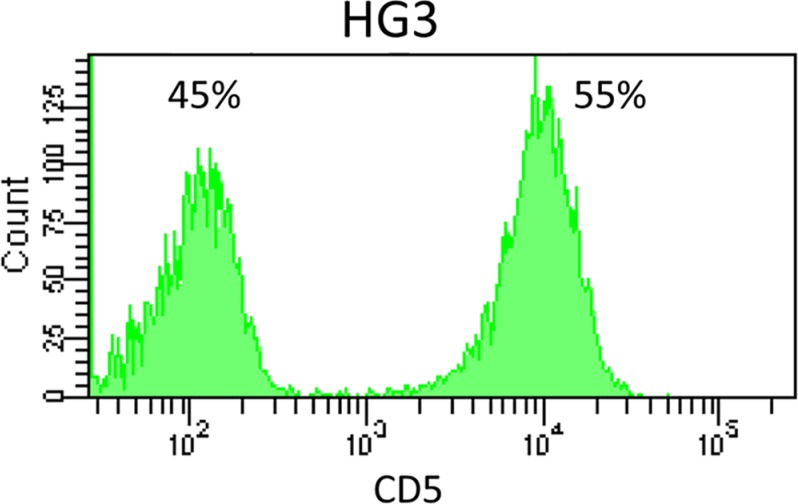
CD5 expression on cell line HG3 Flow cytometry revealed bimodal expression of CD5 in the CLL cell line HG3.

The vast majority of the 284 leukemia and lymphoma cell lines immunophenotyped by us showed rather uniform CD cell surface marker expression patterns, as to be expected from monoclonal cells. However, 12/284 (4.2%) cell lines exhibited bimodal expression of one or several markers (Figure 1, Supplementary Figure S1). Possible explanations for the bimodal cell surface marker expression were: i) *in-vitro* activation leading to the expression of the corresponding markers in a subset of cells, ii) cross-contamination with a second line expressing discordant cell surface markers, or iii) presence of cell line subclones.

To test these competing explanations, we flow-sorted the 12 cell lines with double peaks using antibodies recognizing the corresponding markers (Supplementary Table S2). DNA profiling of the sorted populations revealed that one cell line (WSU-NHL) had been cross-contaminated at source with a second cell line with an as yet undescribed DNA profile. The sorted populations of nine additional cell lines regained bimodal marker expression after 1–2 weeks. We concluded that in these cell lines, bimodal expression was the result of transient activation or differentiation rather than due to subclones.

Cell surface markers remained stable in the sorted subpopulations of the DLBCL cell line U-2932 and the CLL cell line HG3, which were accordingly classified “candidate-biclonal” (Supplementary Table S2).

### Whole exome sequencing identifies cell line subclones

*IG* hypermutation analysis revealed that 6/49 B-lymphoma cell lines with *IG* rearrangement comprised subclones. The stable and differential expression of surface markers suggested that 2/284 cell lines screened might consist of more than one clone. Cell line U-2932 was identified by both techniques. Therefore, we set out to test seven cell lines for mono- or multiclonality.

To verify and molecularly characterize the individual subclones, we single-cell sorted the candidate cell lines. Expression of cell surface markers was used to sort cell lines HG3 (CD5) and U-2932 (CD20, CD38). The other cell lines were single-cell sorted without specific sorting criteria. Clonal growth after sorting was observed in 4/7 cell lines (HG3, SU-DHL-5, TMD-8, U-2932).

We performed whole exome sequencing (WES) of the single-cell cloned cell lines to identify clone-specific mutations as shown in Supplementary Table S3. Results of WES showed that cell lines SU-DHL-5 and U-2932 consisted of 2 subclones while HG3 and TMD-8 comprised three subclones. Expression array analyses revealed that subclones of cell lines HG3 and U-2932 showed abundant differentially expressed genes. Here, we focused on HG3. Cell line U-2932 has been studied previously [5].

### Clonal structure of HG3 subclones

Genetic and epigenetic mechanisms led to the differential expression of more than 100 genes in the U-2932 subclones [5]. Here, we show that the DLBCL cell lines SU-DHL-5 and TMD-8 and the CLL cell line HG3 also comprise subclones. With high numbers of both subclone-specific mutations and differentially expressed genes, cell line HG3 stood out as a potential model for clonal divergence.

WES was performed on four HG3 single cell clones (2 CD5^+^, 2 CD5^−^ clones) showing both shared and clone-specific mutations (Supplementary Figure S2, Supplementary Table S3). *CCND2* was mutated in all clones and belongs to the top 20 genes recurrently mutated in CLL (Supplementary Figure S2A) [9]. The results suggested that the cell line consisted of at least three subclones.

**Figure 2:**
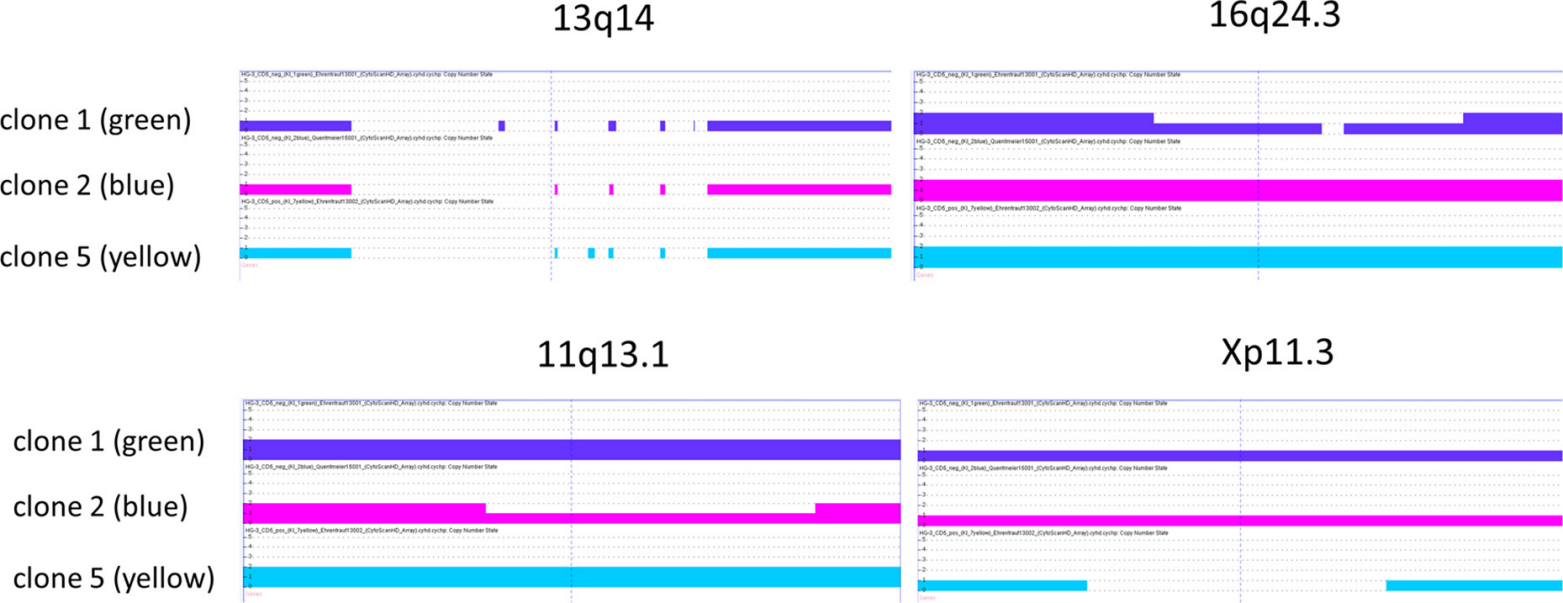
Copy number aberratons of HG3 subclones The deletion in chr13q14 affects *DLEU2*, *DLEU7*, and *miR15/a/miR16-1* and is present in all single cell clones tested. The chr16q24.3 deletion targets *ANKRD11* in clone 1 (green lineage), chr11q13.1 includes *NAA40* (clone 2, blue lineage), chrXp11.3 with *KDM6A* is deleted in clone 5 (yellow lineage).

To verify these subclones at the chromosomal level also, we performed genomic array analyses for three single cell clones. All clones carried the CLL-typical chr. 13q14 deletion, targeting *DLEU2*, *DLEU7*, and *miR15/a/miR16-1* (Figure 2). Confirming their threefold structure, the cell line clones (named for convenience “blue”, “green”, “yellow”) also showed unique, clone-specific numeric aberrations (Figure 2).

These results were achieved with a limited number of single cell clones (WES: 4 clones, Cytoscan array: 3 clones). To test the validity of the conclusion that cell line HG3 comprised three – and not more - clonal lineages, we performed PCR-based amplification-refractory mutation system (ARMS) assays for nine mutated sites and 60 single cell clones. According to WES, these genes were mutant in all or in subsets of the clones. We postulated that the detection of mutations in a large number of clones would inform the clonal structure of the cell line.

**Figure 3:**
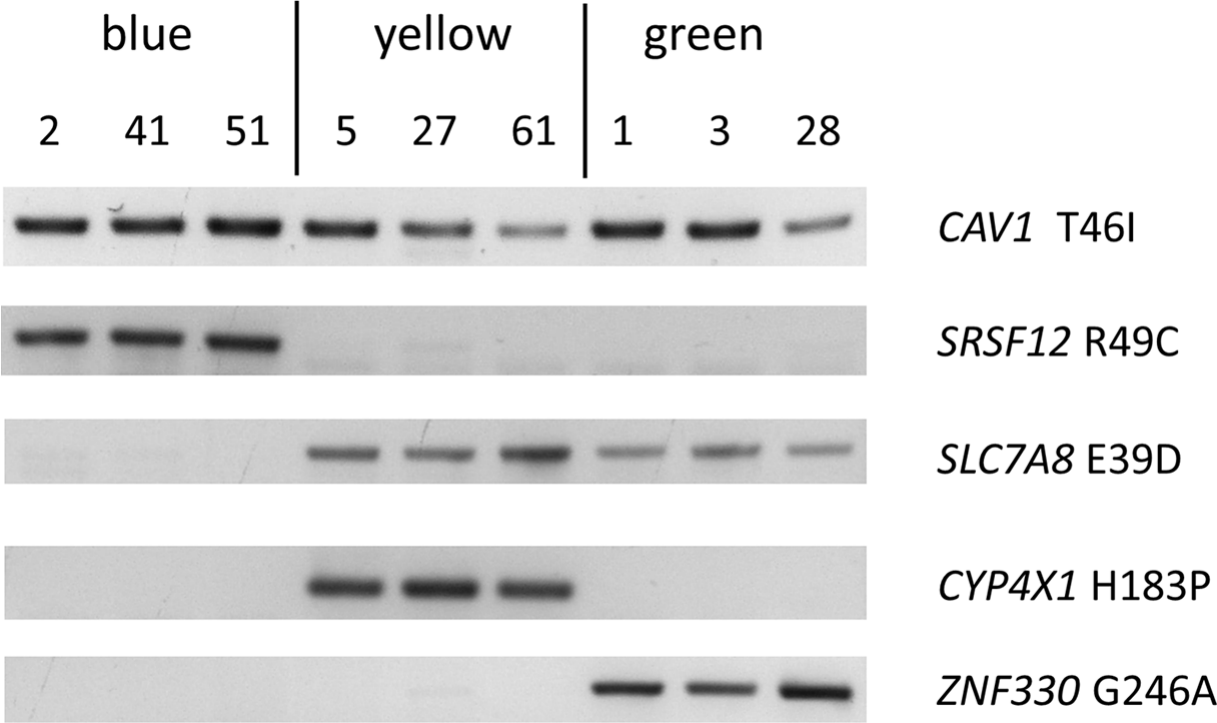
Mutations characterizing subclones of cell line HG3 ARMS assay detecting mutations that are common to all single cell clones (*CAV1* T46I) or subclone-specific. Presence of *SRSF12* R49C and absence of *SLC7A8* E39D identify the blue lineage. Presence of *CYP4X1* H183P and *ZNF330* G246A is characteristic for the yellow and green lineages, respectively.

**Figure 4:**
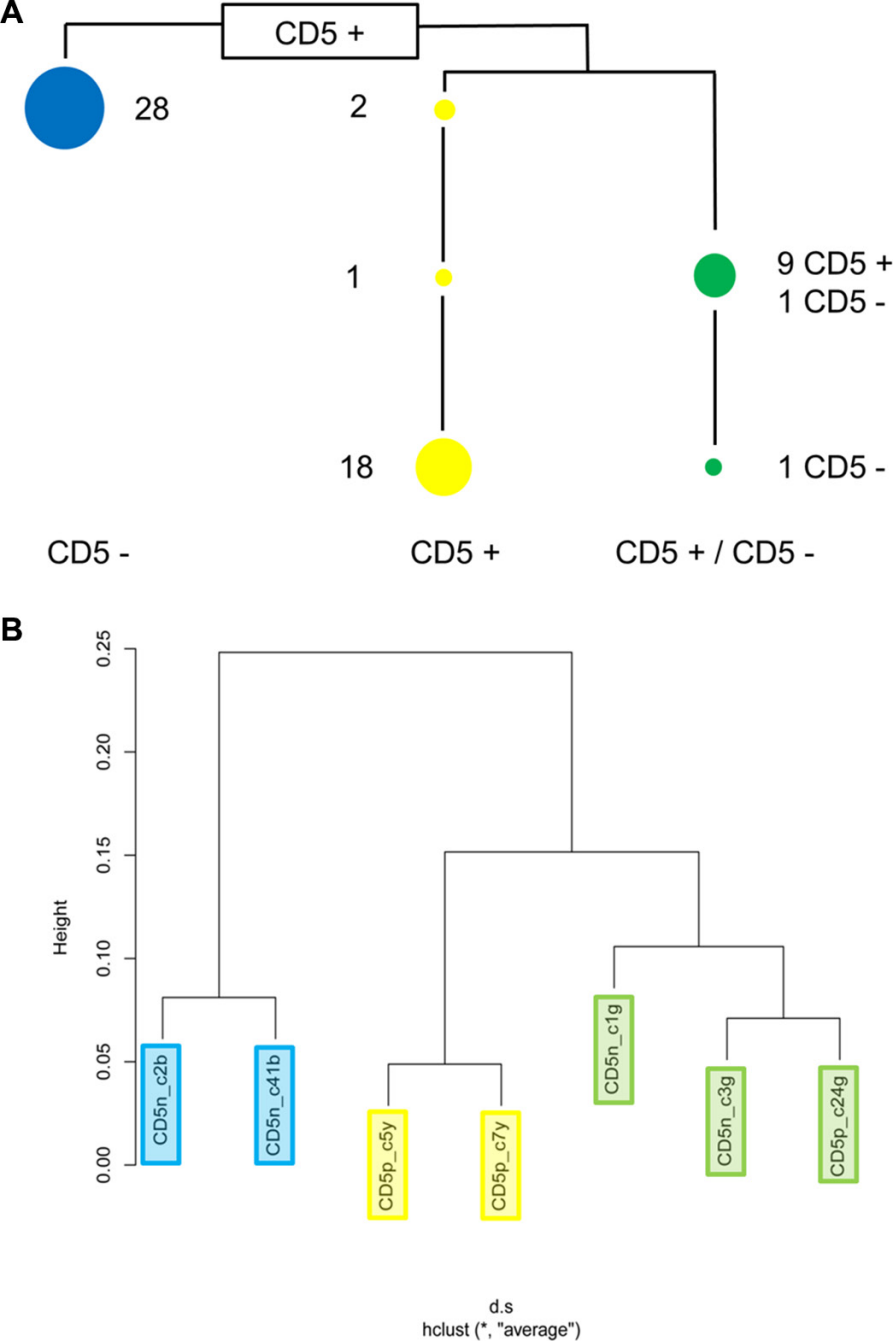
Clonal structure of cell line HG3 (**A**) ARMS assays showing that cell line HG3 comprises three clonal lineages. Numbers indicate how many of the sixty single cell clones tested carry lineage- and stage-specific mutations. The blue lineage is CD5^−^, the yellow lineage is CD5^+^. Single cell clones of the green lineage carrying fewer mutations are CD5^+^, clones with higher numbers of mutations are CD5^−^. Supplementary Table S4 shows the mutational status of the individual single cell clones. (**B**) Unsupervised clustering of expression array data from seven randomly chosen single cell clones revealed the same clonal structure as the mutational analysis.

ARMS assays showed that all 60 clones carried the wild type version of all nine genes tested (Supplementary Table S4). Mutant *CAV1* T46I was found in all 60 clones and thus indicated the putative mother clone (Figure 3, Supplementary Table S4). All other mutations were present only in a subset of clones (partially shown in Figure 3). At least one of five lineage-specific mutations was detected in all 60 cell lines, confirming that cell line HG3 comprised three subclones (Figure 3, Supplementary Table S4). Mutational analysis of four additional sites allowed a more detailled view of their lineage structure (Figure 4A, Supplementary Table S4).

We performed genomic quantitative PCR (qPCR) detecting clone-specific mutations to determine the subclonal composition of cell line HG3 (Supplementary Figure S3). Roughly 50% of the cells belong to the blue lineage, the green and yellow lineages making up the remainder.

### Clone-specific gene expression

**Figure 5:**
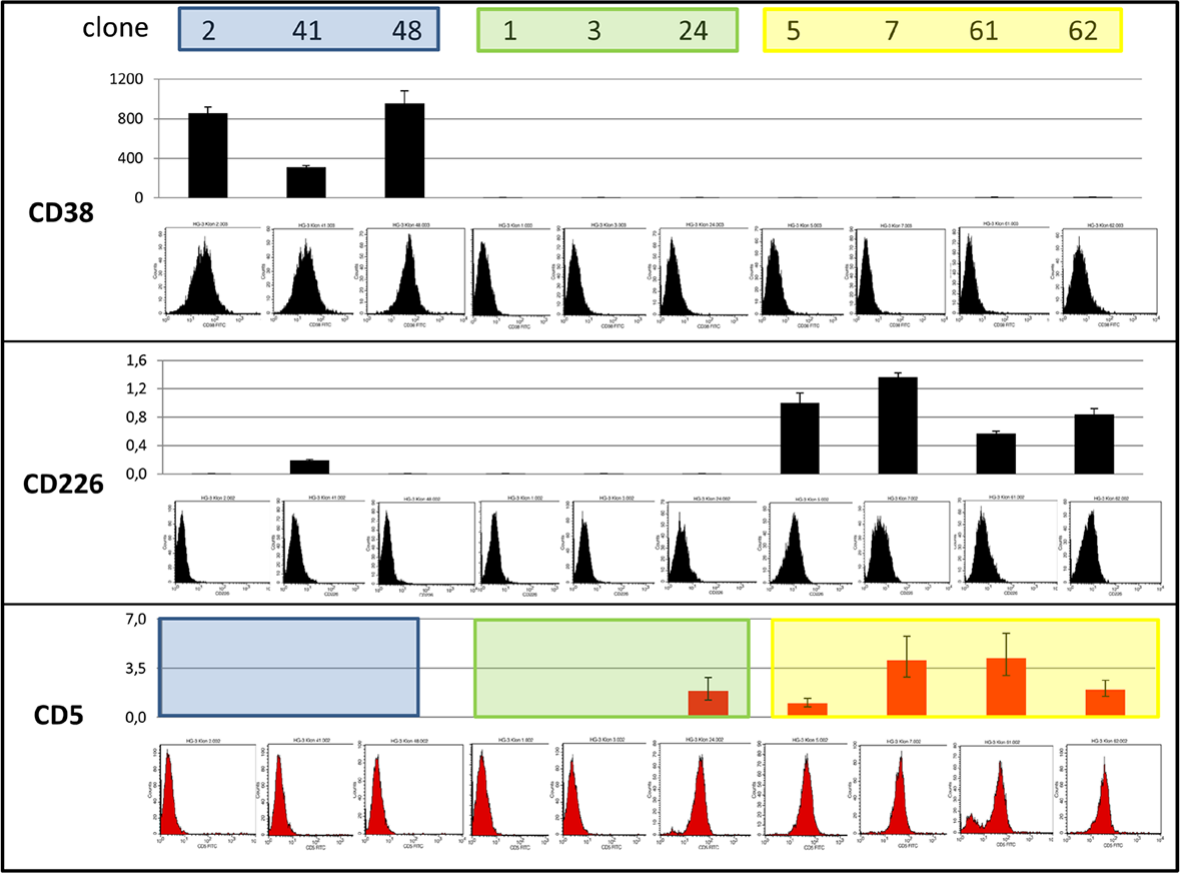
Expression of CD38, CD226 and CD5 According to quantitative reverse-transcriptase PCR (columns) and flow cytometry, clones of the blue lineage were CD38^+^/CD226^−^, green CD38^−^/CD226^−^, and yellow CD38^−^/CD226^+^. Blue clones were CD5^−^, yellow CD5^+^, green CD5^+^ or CD5^−^. One of the yellow clones (clone 61) partially lost CD5 during cultivation.

Unsupervised clustering of gene expression array and methylation array data from seven single cell clones matched the clonal structure of the mutational analysis data (Figure 4, Supplementary Figure S4A). The mRNA and protein expression of the surface antigens CD38 and CD226 distinguished the three clonal lineages, blue CD38^+^/CD226^−^, green CD38^−^/CD226^−^, and yellow CD38^−^/CD226^+^ (Figure 5). However, weak protein expression of CD226 by the cells of the yellow lineage prevented sorting with these markers (Supplementary Figure S5).

**Figure 6:**
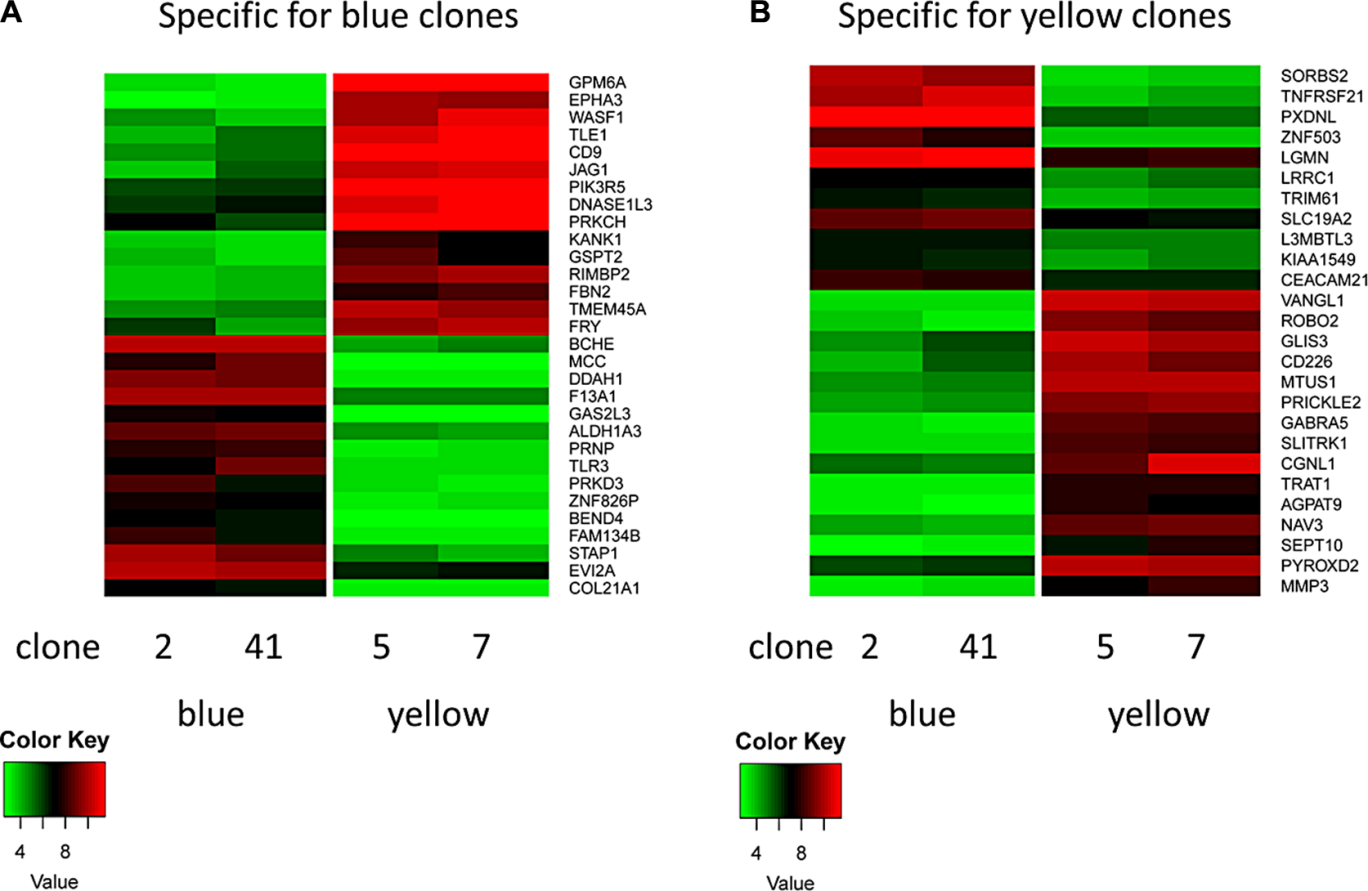
Heatmap of clone-specific genes (**A**) Clones of the blue lineage show a typical set of high (e.g. *BCHE*) and low genes (e.g. *GPM6A*). Clones of the green (not shown) and yellow lineages show the inverse pattern. (**B**) Clones of the yellow lineage show a typical set of high (e.g. *VANGL1*) and low genes (e.g. *SORBS2*). Clones of the green (not shown) and blue lineages show the inverse pattern.

Significant transcriptional differences between the clonal lineages could be found for *CD38* and *CD226* and for a panel of additional genes, e.g. *CD9, GPM6A, SEPT10 and TNFRSF21,* markers or functional mediators in CLL (Figure 6) [10–17].

### CD5 expression in clonal lineages

Cell line HG3 comprised both CD5^+^ and CD5^−^ populations (Figure 1). WES analysis had revealed that both populations consisted of more than a single subclone. FACS analysis of single cell clones helped explain the situation in more detail: cell line HG3 comprised three clonal lineages, one (coded blue) was CD5^−^, the second (yellow) was CD5^+^, and the third, green lineage consisted of CD5^+^ and CD5^−^ clones (Figure 4A, Figure 5). “Early” clones of the green lineage, i.e. those with fewer mutations, were CD5^+^. The “late” clones with more mutations had lost CD5 expression (Figure 4A). Thus, the cell line had lost CD5 twice, during development of the blue lineage and at a later phase of the development of the green lineage.

Loss of CD5 membrane expression has been attributed to a switch from exon 1A, encoding full-length CD5 to exon 1B, which encodes a truncated version of CD5 [6, 7]. We performed reverse transcriptase PCR with a probe detecting *CD5* mRNA independent of the splice status. This analysis revealed that the clones which expressed the protein were also *CD5* mRNA^+^ while clones lacking CD5 on the cell surface, also lacked the corresponding mRNA suggesting that *CD5* was regulated at the transcriptional level (Figure 5). *CD5* showed an inverse correlation between DNA methylation and expression, a signature indicative of epigenetic regulation (Supplementary Figure S4B, Figure 5). Supporting the notion that *CD5* expression was regulated by methylation, treatment with the DNA demethylating agent 5-Aza-2′-deoxycytidine (Aza) led to a 100-fold induction of *CD5* mRNA (Supplementary Figure S6). However, with mRNA levels still 40-fold below untreated CD5^+^ cells, Aza-treated cells still failed to express CD5 on the cell surface (Supplementary Figure S6; protein data not shown). Computational and array-based analyses focussing on potential indirect effects by Aza through regulation of transcription factors failed to resolve this discrcepancy.

Our data suggest that *CD5* is regulated on the level of gene transcription, independent of the alternative splicing reported hitherto. Future studies are planned to elucidate the role of epigenetic mechanisms in this context.

## DISCUSSION

The genetic heterogeneity of tumors is of prime interest for basic and clinical research. Malignant clones evolve under selective pressures, specifically those imposed by therapeutic drugs. We have shown that the DLBCL cell line U-2932 comprises two subclones with different sets of mutations and gene expression patterns [4, 5]. Here, we set out to elucidate how often cell lines consist of subclones.

As evidenced by the presence of specific sets of *IG* mutations, 12% (6/49) of B lymphoma cell lines comprised subclones. Additional biclonal cell lines were detected by subclone-specific expression of cell surface markers. These results showed that subclones in cell lines are not uncommon. We isolated individual subclones to find out whether they can be used as isogenic models for regulatory and functional studies.

Four “candidate multiclonal” cell lines (HG3, SU-DHL-5, TMD-8, U-2932) were successfully single-cell cloned. WES confirmed that cell lines SU-DHL-5 and U-2932 comprised two subclones and cell lines HG3 and TMD-8 three apiece. Due to the lack of archival patient DNA from the other cell lines, subclones could only be traced back to the patient for cell line U-2932. However, the original description of cell line HG3 showed a bimodal curve for the surface expression of CD5, suggesting that the CD5^+^ and CD5^−^ populations of the cell line might also represent subclones of the patient [18]. To find out whether CD5^+^ and CD5^−^ populations represented distinct subclones, we flow-sorted this cell line and performed whole exome sequencing (WES). The sequencing results suggested that CD5^+^ and CD5^−^ populations were themselves polyclonal. Copy number analysis, WES and ARMS analysis of 60 single-cell clones revealed that cell line HG3 comprised three clonal lineages. One subclone was CD5^+^, another was CD5^−^, while the third included “early” CD5^+^ cells with few mutations and “late” CD5^−^ cells with a higher number of mutations.

Unsupervised clustering of gene expression and methylation data both confirmed the clonal structure obtained by mutational analysis. The list of differentially expressed genes included markers or functional mediators in CLL like *CD5, CD9, CD38, GPM6A, SEPT10* and *TNFRSF21* [10–17]. To show that our HG3 subclones could help elucidate how CLL mediators are regulated, we focused on *CD5* as model.

Originally described as a T-cell marker, *CD5* is also expressed on a subset of ‘innate’ (B1) B cells and on CLL cells where, together with CD19^+^, CD20^low^, CD23^+^, CD79^low^, and either kappa or lambda *IG* light chains, they constitute diagnostic criteria [19]. *CD5* physiologically constrains B cell receptor (BCR) signaling in healthy B cells [20]. BCR signaling is also involved in CLL growth and survival where *CD5* regulates selected genes and signaling pathways, thus contributing to CLL development [21–25]. Little has been hitherto reported about the regulation of *CD5* in B cells, whereas in T cells recent data show that *CD5* is developmentally regulated and induced by T cell receptor (TCR) engagement, and furthermore that CD5 surface expression reflects TCR signal intensity and its affinity for positively selecting self-ligands [26]. CD5 expression can fluctuate within the CLL clone from CD5^bright^ to CD5^dim^ depending on its location in the proliferative compartment (lymph node or bone marrow) or in the resting compartment (blood) [27]. Alternatively, the surface antigen may also be lost during disease progression or Richter's transformation of CLL to prolymphocytic leukemia or DLBCL [28, 29]. Loss of CD5 membrane expression has been reported when there is a switch from usage of exon 1A, coding for full-length CD5 that favors anergy, to the use of exon 1B, which encodes a truncated version of CD5, not expressed on the cell surface [6, 7]. We used an array which allowed quantification of *CD5* mRNA independently of the alternative splice variants. Suggesting that *CD5* was regulated at the transcriptional level, *CD5* mRNA was high in CD5^+^ HG3 cells and low in CD5^−^ cells. Supporting the notion that epigenetic/transcriptional events play a major role for *CD5* regulation, *CD5* showed the inverse methylation/expression pattern typical for genes regulated by DNA methylation. These results suggest that epigenetic/transcriptional mechanisms play a role in *CD5* expression independent of the alternative splice variants described so far. Future studies will address whether DNA-demethylating agent Aza (100× increase of *CD5* mRNA) directly affected *CD5* or indirectly, e.g. by transcription factor stimulation.

In conclusion, a substantial number of *IG* hypermutated B-lymphoma cell lines comprise subclones (6/49; 12%). We have shown that bimodal expression of cell surface markers may be used to detect the presence of subclones, independent of *IG* hypermutations. Both screening methods require additional assays like WES to verify subclones. Archival patient DNA is obligatory to determine whether cell line subclones truly represent tumor clones.

We describe the detailed clonal structure of the CLL cell line HG3 and elucidate loss of *CD5* expression in 2/3 subclones. We were able to show that *CD5* can be regulated at the epigenetic/transcriptional level rather than by the alternative splicing mechanism described hitherto – further demonstrating the benefit of isogenic subclones for studying gene regulation.

We trust that our findings will encourage other scientists to test “early” batches of their cell lines for subclones, as these may prove useful isogenic models for regulatory and functional studies, notably the potential impact of clonal heterogeneity on therapeutic response. Moreover, we urge cryopreservation of early cell line passages together with uncultured patient material and their reposition with facilities, such as cell banks, to enable investigation of clonal structure of different cancers using whole genome sequencing methods when these become routinely applicable.

## MATERIALS AND METHODS

### Cell lines

Cell lines were taken from the stock of the cell lines bank (DSMZ – German Collection of Microorganisms and Cell Cultures). Others were obtained “for research purpose only”. Cell lines were authenticated by DNA profiling and cytogenetics. Detailed references and cultivation protocols have been described previously [30].

### Screening for immunoglobulin rearrangements and immunoglobulin hypermutations

Rearrangements of *IG* heavy chains in B cell lines were determined by PCR using VH- and JH primers described by van Dongen [8] (Supplementary Table S5). To check for hypermutations, PCR products were cloned into the pGEM-TEasy vector (Promega, Madison, WI, WSA) and sequenced.

### Flow cytometry

Single cell sorting was performed on a FACSAriaIII (Becton Dickinson, Heidelberg, Germany). APC-labeled CD5 antibody (Ab) clone UCHT2 (Becton Dickinson) was used to sort cell line HG3. PerCP-conjugated CD20 and APC-conjugated CD38 (Becton Dickinson) were used to sort U-2932 populations. For phenotypic analysis of HG3 subclones, we applied PE-conjugated CD38 and FITC-conjugated CD226 Abs (Becton Dickinson). Growth of sorted cell clones was observed over a four-week period by microscopy. Clones were harvested and subjected to expression array analysis and WES.

### Numerical aberrations

CytoScan HD Array (Affymetrix, Santa Clara, CA, USA) hybridization analysis was performed to identify numerical aberrations. DNA was prepared using the Qiagen Gentra Puregene Kit (Qiagen, Hilden, Germany). Data were analyzed using the Chromosome Analysis Suite software version 2.0.1.2 (Affymetrix).

### Whole exome sequencing

The concentration and quality of the purified genomic DNA (gDNA) was determined by an Agilent Technologies 2100 Bioanalyzer (Agilent Technologies; Waldbronn, Germany). Fragmentation of 100 ng gDNA in 55 μl Tris-EDTA buffer in a microtube (Covaris) was performed on a Covaris S2 (duty cycle 10%, intensity 4-5, 200 cycles per burst during 110-360 s) to obtain fragments with an average length of 120–200 base pairs (bp). Fragment size was checked with an Agilent Technologies 2100 Bioanalyzer. The DNA sequencing library was generated from 100 ng of fragmented gDNA using Agilent SureSelectXT Reagent Kits v5 (50 Mb) and SureSelectXT Reagent Kits v5_UTR (75 Mb) according to the manufacturer's protocols. The final DNA sequencing library was purified, size controlled by Agilent Technologies 2100 Bioanalyzer (High Sensitivity DNA Chip) and prepared for sequencing according to the manufacturer's protocol (Illumina). The libraries were sequenced on Illumina HiSeq2500 using TruSeq SBS Kit v3-HS (200 cycles, paired end run) with 25.6-44.4 million reads per sample resulting in > 50× mean coverage. Reads were trimmed for poor quality and adapter/primer sequences (ea-utils 1.1.2-686), mapped to the hg19/GRCh37 genome annotation (STAR 2.4.0b), sorted and converted (samtools 0.1.19), sequence duplicates removed (picard 1.121) and subsequently variants were identified via GATK (3.3-0) and VarScan (1.1.2-686) tools and mutation effects revealed via Ensembl VEP (release 77) [31, 32]. Overlapping mutations of GATK and VarScan were selected for further analyses and filtered to > 20×depth, > 0.25% allele frequency, to missense mutations and to < 0.05 strand bias. WES data of cell lines SU-DHL-5 and TMD-8 are deposited under entry number E-MTAB-4956 (Array-Express), data of cell lines HG3 and U-2932 under E-MTAB-4527.

### ARMS assay and real-time PCR

ARMS assay, a PCR-based mutational analysis assay, was performed for nine mutations detected by WES to determine the clonal structure of cell line HG3. Primers are listed in Supplementary Table S6. Quantiative ARMS PCR was performed on a 7500 Applied Biosystems (Darmstadt, Germany) real-time PCR system using the SYBR green assay (Applied Biosystems) with *ABL1* as internal control. Relative expression levels were calculated using the 2^^^-DDCt-method.

### DNA microarray hybridization

500 ng total RNA were used for biotin labelling according to the 3′ IVT Express Kit (Affymetrix). 7.5 μg of biotinylated cRNA were fragmented and placed in a hybridization cocktail containing four biotinylated hybridization controls (BioB, BioC, BioD, and Cre). Samples were hybridized to an identical lot of Affymetrix GeneChip HG-U133 Plus 2.0 for 16 h at 45°C. Steps for washing and SA-PE staining were processed on the fluidics station 450 using the recommended FS450 protocol (Affymetrix). Image analysis was performed on GCS3000 Scanner and GCOS1.2 Software Suite (Affymetrix). Analysis of data was performed using GeneSpring 11.5.1 (Agilent Technologies; Santa Clara, CA, USA). After RMA-background correction and quantile normalization of spot intensities, data were further processed by division to the sample mean and logarithmic transformation for centering the values around zero. Data processing was done via R/Bioconductor using limma and affy packages [33, 34]. Expression array data of HG3 subclones are deposited under entry number E-MTAB-4955 (Array Express).

### Gene expression analyses

RNA was prepared using the RNeasy Mini kit (Qiagen). For mRNA quantification, reverse transcription was performed using the SuperScript II reverse transcriptase kit (Invitrogen, Karlsruhe, Germany). TaqMan probes (Applied Biosystems) were used to quantify human *CD5* (Hs 00204397_m1), *CD38* (Hs 01120071_m1), *CD226* (Hs 00170832_m1) expression levels with *TBP* as endogenous control. Relative expression levels were calculated using the DDCt method.

### Array-based DNA-methylation analysis

For DNA-methylation analysis the Infinium HumanMethylation450 BeaChip (Illumina) was used according to the manufacturer's instruction. This platform allows the interrogation of 485,577 assays (482,421 sites, 3,091 non-CpG sites, 65 random SNPs) in parallel at a single-nucleotide resolution per sample [35]. Arrays were scanned using the Illumina iScan. Raw hybridization signals were processed using the GenomeStudio software (version 2011.1; Methylation analysis Module version 1.9.0, Illumina) applying the default settings and internal controls for normalization. The threshold for the detection *p*-value was set to < 0.01 per CpG site.

### Inhibitors

Aza (Sigma) dissolved in DMSO was used to verify the effect of methylation on expression of *CD5*. Cells were seeded at a density of 5 × 10^5^ cells/ml, Aza was added at a final concentration of 5 μM. Control cells were treated with 0.03% DMSO. After 2 d, half of the medium was replenished with medium with/without Aza (5 μM). After 3 d, cells were harvested to prepare RNA.

## SUPPLEMENTARY MATERIALS FIGURES AND TABLES












