# Supplementary material for: Subclones in B-lymphoma cell lines: isogenic models for the study of gene regulation
Source: Oncotarget. 2016 Aug 23;7(39):63456–65. doi: 10.18632/oncotarget.11524 (PMC5325377; doi:10.18632/oncotarget.11524)
Supplement: Supplementary file 1 [file oncotarget-07-63456-s001.pdf]

Subclones in B-lymphoma cell lines: isogenic models for the study of gene regulation

Supplementary Materials

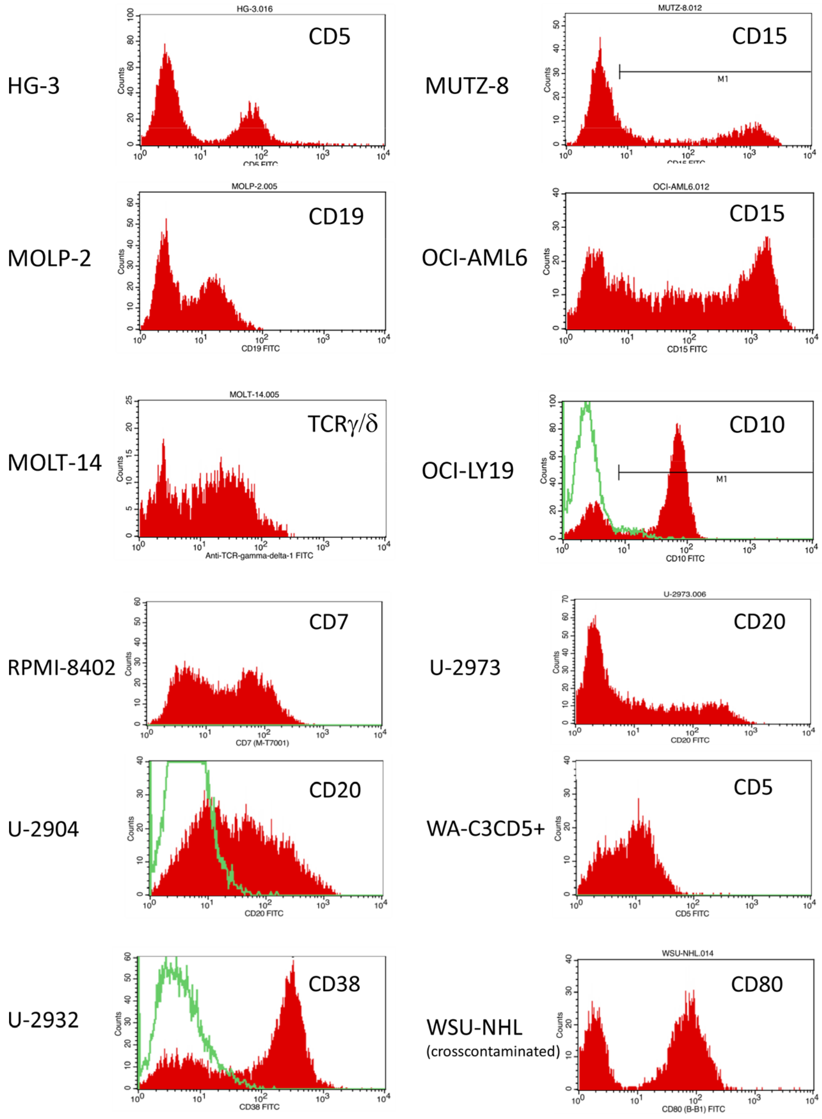

Supplementary Figure S1: Bimodal surface markers expression. Flow cytometry revealed bimodal expression surface markers on 12 cell lines of various hematopoetic lineages. The ratio of the individual populations (marker^{pos} and marker^{neg}) is not always the same in different batches, indicating that the size of the populations is variable.

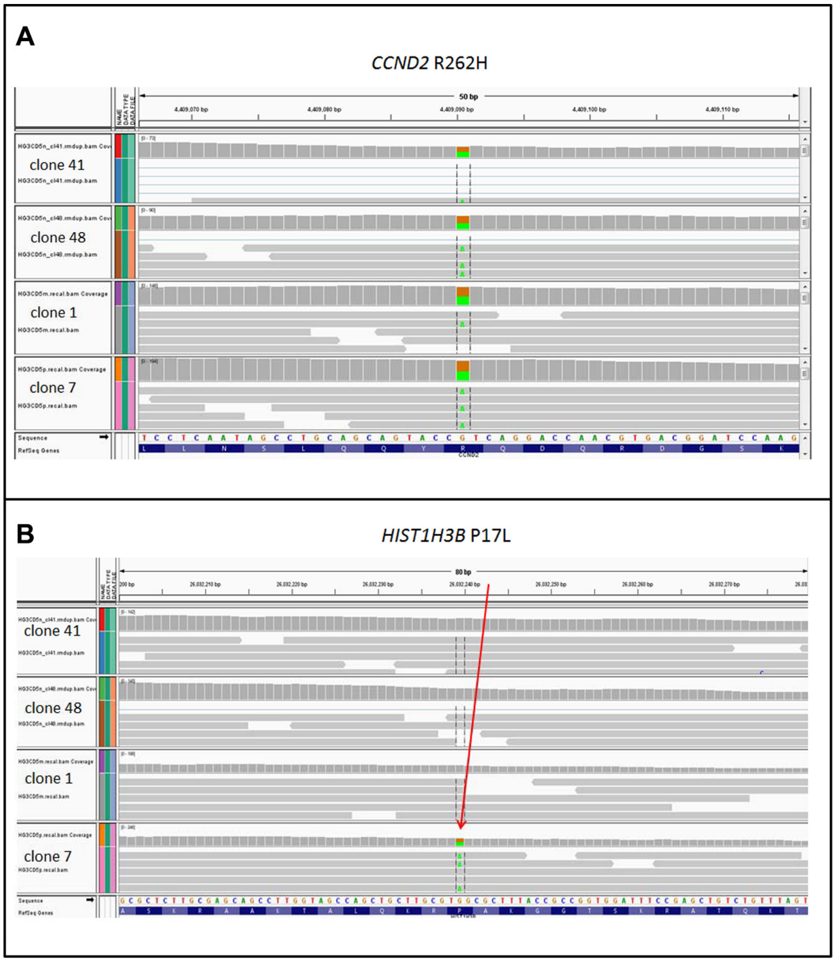

Supplementary Figure S2: WES results. (**A**) The *CCND2* R262H mutation was detected in all four single cell clones sequenced. The variant had been described as rare SNP (rs 142170178; MAF < 0.01). (**B**) The *HIST1H3B* P17L mutation (arrow) was found in one of the four single cell clones.

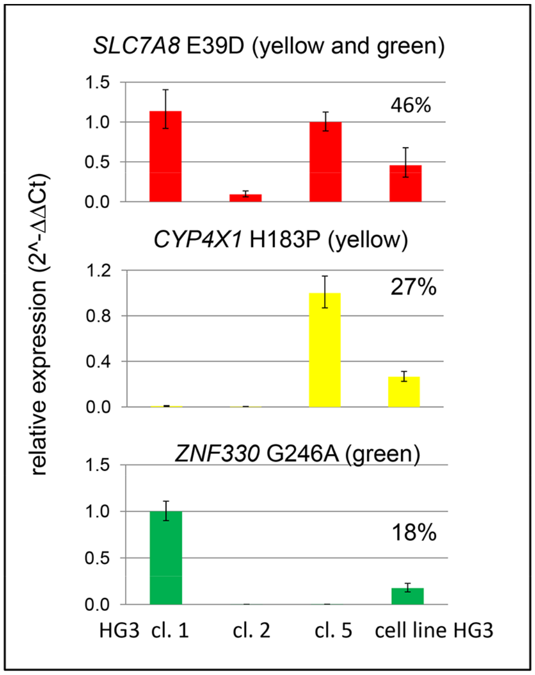

Supplementary Figure S3: Lineage-specific quantitative genomic PCR. Genomic qPCR was performed to assess the clonal composition of cell line HG3. Single cell clones carrying a specific mutation were used as reference.

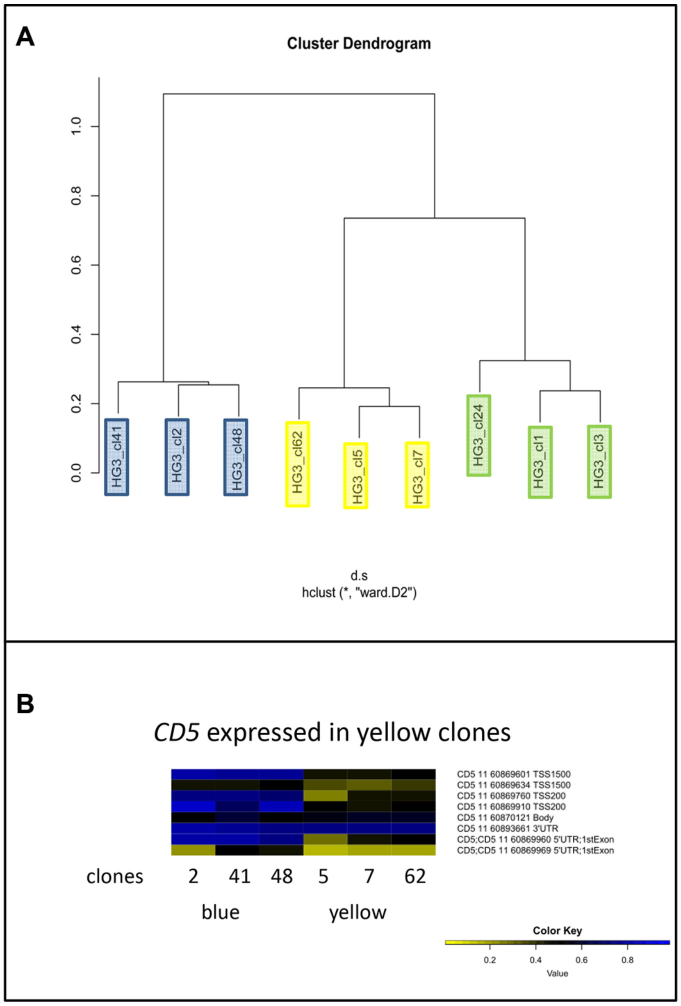

Supplementary Figure S4: DNA methylation analysis. (**A**) Methylation analysis reveals the same clonal structure of cell line HG3 as mutational analysis and expression array analysis (see Figure 4A, 4B). (**B**) Yellow clones are $CD5^{+}$, blue clones are $CD5^{-}$. Repression is accompanied by DNA methylation. Color code: blue (high methylation), yellow (low methylation).

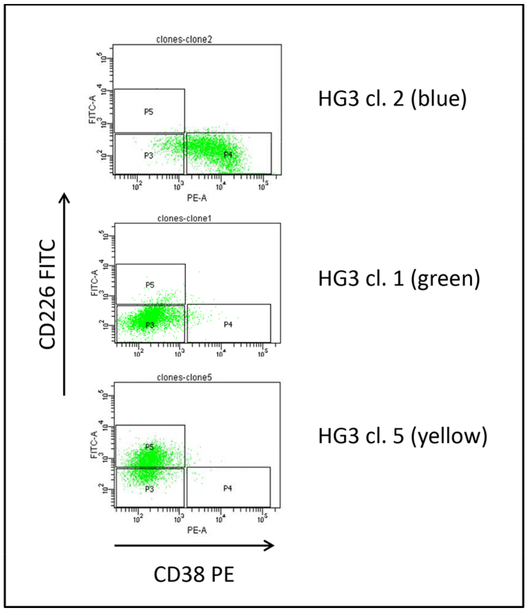

Supplementary Figure S5: CD38 and CD226 expression on clones of cell line HG3. Expression of surface antigens was determined using PE-labeled CD38 and FITC-labeled CD226 Abs.

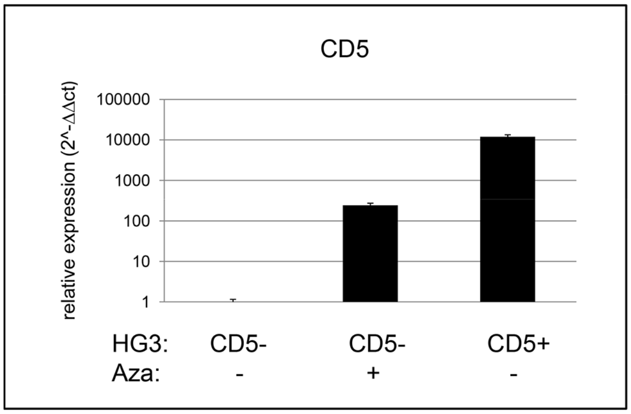

Supplementary Figure S6: CD5 expression after DNA demethylation. *CD5* mRNA expression of HG3 cells (CD5- population) was assessed by qPCR. Aza (5 µM) was given for 72 h.

Supplementary Table S1: Ig hypermutation of B-lymphoma cell lines. See Supplementary_Table_S1

Supplementary Table S2: Cell lines with bimodal surface markers. See Supplementary_Table_S2

Supplementary Table S3: Subclone-characteristic mutations. See Supplementary_Table_S3

Supplementary Table S4: Clonal composition of cell line HG3. See Supplementary_Table_S4

Supplementary Table S5: Sequence of Ig rearrangement primers

VH1	forward	5′- GGC CTC AGT GAA GGT CTC CTG CAA G - 3′
VH2	forward	5′- GTC TGG TCC TAC GCT GGT GAAA ACC C - 3′
VH3	forward	5′- CTG GGG GGT CCC TGA GAC TCT CCT G - 3′
VH4	forward	5′- CTT CGG AGA CCC TGT CCC TCA CCT G - 3′
VH5	forward	5′- CGG GGA GTC TCT GAA GAT CTC CTG T -3′
VH6	forward	5′- TCG CAG ACC CTC ACT CAC CTG TG - 3′
JH concensus	reverse	5′- CTT ACC TGA GGA GAC GGT GAC C - 3′

Supplementary Table S6: Sequence of ARMS primers. See Supplementary_Table_S6
